# Supplementary material for: In-hospital stress and patient outcomes: A systematic review and meta-analysis
Source: PLoS One. 2023 Mar 9;18(3):e0282789. doi: 10.1371/journal.pone.0282789 (PMC9997980; doi:10.1371/journal.pone.0282789)
Supplement: S1 File — (DOCX) [file pone.0282789.s001.docx]

Appendix A

**Search terms, last conducted 2^nd^ February 2023:**

Table 1. Search terms using MeSH in the MEDLINE database (OvidSP)

| **Search** | **PICO** | **Query** | **Results** |
| --- | --- | --- | --- |
| #1 | Population | (Inpatients/) OR (Hospitalization/) | 156,626 |
| #2 | Exposure | (Stress, Psychological/) | 132,612 |
| #3 | Outcome  (MeSH) | (exp Hospitalization/) OR (exp Outcome Assessment, Health Care/) OR (exp Postoperative Complications/) OR (exp Iatrogenic Disease/) OR (exp Vital Statistics/) OR (exp Pain/) OR (exp Infections/) OR (Patient Satisfaction/) OR (Quality of Life/) OR (Quality-Adjusted Life Years/) | 5,980,766 |
| #4 | Outcome  (keyword) | (post-hospital syndrome or posthospital syndrome).mp. | 38 |
| #5 |  | #3 OR #4 | 5,980,773 |
| #6 |  | #1 AND #2 AND #5 | 1066 |
| #7 |  | Limit #4 to (Abstracts, English Language, Human, “All Adults (19 plus years)”) | 524 |

Table 2. Search terms using Emtree in the EMBASE database (OvidSP)

| **Search** | **PICO** | **Query** | **Results** |
| --- | --- | --- | --- |
| #1 | Population | (Hospital Patient/) OR (Hospitalization/) | 696,732 |
| #2 | Exposure | (Stress/) OR (Acute Stress/) OR (Behavioural Stress/) OR (exp Chronic Stress/) OR (Emotional Stress/) OR (Interpersonal Stress/) OR (Mental Stress/) OR (Social Stress/) | 306,465 |
| #3 | Outcome  (Emtree) | (Hospital Readmission/) OR (Length of Stay/) OR (exp Treatment Outcome/) OR (exp Postoperative Complication/) OR (exp Iatrogenic Disease/) OR (exp Mortality/) OR (Morbidity/) OR (exp Pain/) OR (exp Infection/) OR (Patient Satisfaction/) OR (exp Quality of Life/) | 9,743,282 |
| #4 | Outcome  (keyword) | (post-hospital syndrome or posthospital syndrome).mp. | 55 |
| #5 |  | #3 OR #4 | 9,743,292 |
| #6 |  | #1 AND #2 AND #5 | 2649 |
| #7 |  | Limit #4 to (Abstracts, English Language, Human, (Adults or Aged), (Article or Article in press)) | 832 |

Table 3. Search terms using the PsycINFO Thesaurus in PsycINFO (OvidSP)

| **Search** | **PICO** | **Query** | **Results** |
| --- | --- | --- | --- |
| #1 | Population | (exp Patients/) OR (Hospitalization/) | 111,960 |
| #2 | Exposure | (Stress/) OR (Chronic Stress/) OR (Environmental Stress/) OR (Psychological Stress/) OR (Social Stress/) | 88,618 |
| #3 | Outcome  (Thesaurus) | (Hospital Admission/) OR (exp Treatment Outcomes/) OR (Health Outcomes/) OR (Postsurgical Complications/) OR (exp Physical Health Assessment/) OR (Death and Dying/) OR (Morbidity/) OR (exp Pain/) OR (exp Infectious Disorders/) OR (exp Health Status/) OR (Treatment Effectiveness Evaluation/) | 402,478 |
| #4 | Outcome  (keyword) | (post-hospital syndrome or posthospital syndrome).mp. | 5 |
| #5 |  | #3 OR #4 | 402,481 |
| #4 |  | #1 AND #2 AND #5 | 219 |
| #5 |  | Limit #4 to (Abstracts, English Language, Human, Adulthood, Journal) | 130 |

Table 4. Search terms using CINAHL Subject Terms in CINAHL (EBSCO)

| **Search** | **PICO** | **Query** | **Results** |
| --- | --- | --- | --- |
| #1 | Population | Used the *inpatient* search filter | N/A |
| #2 | Exposure | (MH “Stress”) OR (MH “Stress, Psychological”) | 69,251 |
| #3 | Outcome  (Subject Terms) | (MH “Institutionalization+”) OR (MH “Outcomes (Health Care)+”) OR (MH “Postoperative Complications+”) OR  (MH "Iatrogenic Disease") OR (MH “Vital Statistics+”) OR (MH "Pain+") OR (MH “Infection+”) OR (MH “Patient Satisfaction”) OR (MH “Quality of Life”) OR (MH “Quality-Adjusted Life Years”) | 1,488,253 |
| #4 | Outcome (keyword) | Post hospital syndrome | 32 |
| #5 |  | #3 OR #4 | 1,488,269 |
| #6 |  | #1 AND #2 AND #5 | 471 |
| #7 |  | Limit #4 to (Abstract Available, English Language, Human, All Adult, Peer Reviewed, Research Article) | 155 |

Table 5. Search terms used for navigating Web of Science

| **Search** | **PICO** | **Query** | **Results** |
| --- | --- | --- | --- |
| #1 | Population | TS = (*hospital* AND inpatient*) | 75,965 |
| #2 | Exposure | TS = (*stressor* OR “stress” NOT (“stress incontinence” OR “stress urinary” OR “oxidative stress” OR “*traumatic stress”)) | 1,731,293 |
| #3 | Outcome | TS = (readmission* OR readmit* OR “length of stay” OR “patient outcome*” OR “treatment outcome*” OR “surgery outcome*” OR complication* OR “iatrogen*” OR mortality OR morbid* OR pain* OR infect* OR “patient satisf*” OR “quality of life” OR “quality-adjusted life years” OR “post-hospital syndrome” OR “posthospital syndrome”) | 5,531,768 |
| #4 |  | #1 AND #2 AND #3 | 677 |
| #5 |  | Limit #4 to (Languages: English; Document Types: Articles) | 586 |

TOTAL (before removing duplicates): 2227

TOTAL (after removing duplicates): 2035

Appendix B

EPHPP Assessments for each of the included studies (1 – strong; 2 – moderate; 3 – weak)

| **Author, year** | **A** | **B** | **C** | **D** | **E** | **F** | **Overall** |
| --- | --- | --- | --- | --- | --- | --- | --- |
| Ahmadi, 1985 | 3 | 3 | 2 | 1 | 1 | 2 | 3 |
| Baharlooei et al., 2017 | 3 | 3 | 1 | 2 | 1 | 2 | 3 |
| Chalageri et al., 2021 | 3 | 2 | 2 | 3 | 1 | 1 | 3 |
| Edmondson et al., 2014 | 3 | 2 | 2 | 3 | 3 | 1 | 3 |
| Karademas et al., 2009 | 2 | 3 | 2 | 1 | 3 | 2 | 3 |
| Karaer et al., 2021 | 3 | 3 | 2 | 3 | 2 | 3 | 3 |
| Pati et al., 2016 | 2 | 1 | 2 | 2 | 1 | 1 | 1 |
| Tully et al., 2008 | 2 | 3 | 2 | 2 | 1 | 1 | 2 |
| Tully et al., 2011 | 2 | 3 | 2 | 2 | 1 | 1 | 2 |
| Volicer, 1978 | 2 | 3 | 1 | 2 | 2 | 2 | 2 |

Appendix C

#### Start-up# ###

# Load packages

library(dplyr)

library(metafor)

library(robumeta)

library(weightr)

# Set WD (change this to wherever your data is stored)

setwd("~/OneDrive - University of Leeds/YQSR work/Dan Ford review/Code and data")

# Import data

newmeta <- read.csv('newmeta.csv')

#### Meta ####

# Convert rs to zs for meta

dat <- escalc(measure = "ZCOR", ri=r, ni=n, data = newmeta, slab = paste(authors, year, sep = ", "))

# Random effects meta-analysis results

res <- rma(yi, vi, data = dat)

# 95% CI for heterogeneity stats

confint(res)

# Back-transform meta-z to r, then produce overall pooled effect

predict(res, digits = 3, transf = transf.ztor)

# Forest plot of results

forest(res, xlim = c(-.7,1), ilab = dat$outcome, ilab.xpos = -.35, atransf = transf.ztor, at=transf.rtoz(c(-.2,0,.2,.4,.6), digits=c(2,1)))

text(-.63, 17, cex=1.5, "Author, year")

text(-.35, 17, cex=1.5, "Outcome")

text(.9, 17, cex=1.5, "Correlation [95% CI]")

text(-.49, -.9, bquote(paste(":  (Q = ", .(formatC(res$QE, digits=2, format="f")), ", df = ", .(res$k - res$p), ", p = ", .(formatC(res$QEp, digits=2, format="f")), "; ", I^2, " = ", .(formatC(res$I2, digits=1, format="f")), "%)")))

#### Robustness ####

# Assess influence of individual studies

influence(res)

# Assess pooled effects in leave one out analyses

leave1out(res)

# Assess small study bias visually (asymmetry)

funnel(res)

# Assess asymmetry statistically in three ways (3rd method good for outliers)

regtest(res)

ranktest(res)

weightfunct(dat$yi, dat$vi, table = TRUE)

# Assess robustness of results to omitted small studies, then visualise potential asymmetry

trimfill(res)

funnel(taf)

# Reproduce results but with robust variance estimation (RVE) meta-analysis

robu(formula = yi ~ 1, data = dat, studynum = sample, var.eff.size = vi, modelweights = "HIER", small = TRUE)

### Sub-group analyses ###

# Study quality (0 = poor; 1 = strong/moderate)

rma(yi, vi, subset = quality == 0, data = dat)
rma(yi, vi, subset = quality == 1, data = dat)

# Patient outcome measure (0 = objective; 1 = subjective (self-reported))

rma(yi, vi, subset = self == 0, data = dat)
rma(yi, vi, subset = self == 1, data = dat)

# Patient outcome measure (0 = post-hospital; 1 = in-hospital)

rma(yi, vi, subset = hospital == 0, data = dat)
rma(yi, vi, subset = hospital == 1, data = dat)

#### Meta-regression analyses ####

# Individual predictor effects

rma(yi, vi, mods = ~ age, data = dat) # Age - continuous

rma(yi, vi, mods = ~ male, data = dat) # Gender - male versus female (assuming 1 = male)

rma(yi, vi, mods = ~ quality, data = dat) # Quality - Good versus bad (assuming 1 = good)

rma(yi, vi, mods = ~ self, data = dat) # Measure type - Objective versus subjective (assuming 1 = objective)

rma(yi, vi, mods = ~ hospital, data = dat) # Outcome type - In-hospital versus out-hospital (assuming 1 = In-hospital)

# Effects of all variables, adjusting for the effects of all the others

rma(yi, vi, mods = ~ age + male + quality + self + hospital, data = dat)
