# Supplementary material for: In-hospital stress and patient outcomes: A systematic review and meta-analysis
Source: PLoS One. 2023 Mar 9;18(3):e0282789. doi: 10.1371/journal.pone.0282789 (PMC9997980; doi:10.1371/journal.pone.0282789)
Supplement: Attachment — Submitted filename: Response to Reviewers.docx [file pone.0282789.s003.docx]

6^th^ February 2023

Dear Dr. Abdelbasset,

**Manuscript ID: PONE-D-22-19657**

**In-hospital stress and patient outcomes: A systematic review and meta-analysis**

Thank you for providing us with detailed feedback for our paper. We have addressed each of the points raised by Reviewers #1 and #2 and believe that this has added further clarification within the method and results sections. We hope that you feel we have satisfactorily responded to the points raised or have provided sufficient justification where an alternative approach has been taken. We feel that the manuscript is now suitable for publication in PLOS ONE. However, we will be very happy to respond to any additional comments.

For your reference, we have addressed each of the reviewers’ comments below, and have provided detail on where and how we have addressed each comment.

**Reviewer #1:**

This is a systematic review and meta-analysis of the existing evidence between in-hospital stress and patient outcomes.
The topic of this manuscript is interesting. The paper is well written and straightforward.
I have some major concerns
1. The authors explained the concept of in-hospital stress and patient outcomes in the introduction, which I appreciated. However, I am not convinced about the relevancy to merge atrial fibrillation in the other outcomes. The atrial fibrillation was the only real medical complication that was included in these studies. Even I understand that comes from a study designed for atrial fibrillation, I found this outcome is really different from the others.

**Response:** We agree with the reviewer and appreciate that *incidence of atrial fibrillation* is different to the other outcome measures. As a result, we have added a comment on page 21 that acknowledges this issue. Moreover, it is worth noting that this study met the review inclusion criteria and therefore we think it appropriate to leave in the meta-analysis. However, we have conducted a leave-one-out analysis (see page 19) to ensure that inclusion of this study (or any other study) is not having a disproportionate influence on our results. To this end, we have stated that:

“Systematically removing one of the 15 correlations at a time, via leave-one-out analysis, indicated that no single effect size was disproportionately contributing to the model. The pooled effect size ranged from: *r* = 0.173 (-0.018) to *r* = 0.205 (+0.014), with each model remaining significant (*p* < 0.001)”.

2. The authors said that the post-hospital syndrome was not a potential heading or a keyword for the databases. Did they require the assistance of a librarian? Although there is no specific heading, it should be possible to look for this concept in different databases. For example, in CINAHL, one could use (hospital-associated N3 (decline OR function* OR decondition* OR Deterioration))
OR (post-discharge N3 (decline OR function* OR decondition* OR cognit* OR Deterioration))
I am surprised that there was no studies with the post hospital syndrome.

**Response:** We have re-run the searches including ‘post-hospital syndrome’ and ‘posthospital syndrome’ as keywords (see S1_Appendices.docx). This also allowed us to update the search from July 2022 to February 2023. This yielded 113 further papers to screen (after de-duplication), but no additional studies were eligible for inclusion in the systematic review and meta-analysis. We have updated search result numbers in-text (pages 2 & 11) and within the PRISMA flow diagram (page 12). We have also updated the search term justification (page 8) to include the new keyword.

The reason for this absence of additional studies is due to the post-hospital syndrome (PHS) literature typically measuring PHS exposure as a hospital admission [30 days / 90 days / one year] prior to the current hospital admission (e.g. Hart et al., 2020; Kirshenbaum et al., 2019; Lim et al., 2020; Sharoky et al., 2017). In our review, we wished to look more specifically at the level of stress experienced in hospital, rather than the mere presence of a hospital admission (which would be a different review entirely).

Additionally, we thank the reviewer for their elegant solution to include the effects of post-hospital syndrome in the search without the specific heading. However, re-running the CINAHL search with the two examples yielded no additional papers.

Hart, S. T., Nelson, M., Kirshenbaum, E., Chen, Y., Mueller, E. R., & Gupta, G. (2020). Post-hospital syndrome predicts poor postoperative outcomes and increased cost following transvaginal midurethral sling placement. *International Urogynecology Journal*, *31*, 1417-1422.

Kirshenbaum, E. J., Nelson, M., Hehemann, M. C., Kothari, A. N., Eguia, E., Farooq, A., ... & Santos, G. D. (2019). Impact of post-hospital syndrome on penile prosthesis outcomes: a period of global health risk. *The Journal of Urology*, *201*(1), 154-159.

Lim, S., Alarhayem, A. Q., Farivar, B., Smolock, C. J., Kirksey, L., Caputo, F. J., ... & Hardy, D. M. (2020). Impact of posthospital syndrome on outcomes of elective endovascular repair of abdominal aortic aneurysm. *Journal of Vascular Surgery*, *72*(5), 1618-1625.

Sharoky, C. E., Collier, K. T., Wirtalla, C. J., Sinnamon, A. J., Neuwirth, M. G., Kuo, L. E., ... & Kelz, R. R. (2017). Hospitalization in the year preceding major oncologic surgery increases risk for adverse postoperative events. *Annals of Surgical Oncology*, *24*, 3477-3485.

Here are some minor concerns:
1. In the flow-chart, there was three records identified through other sources, please specify what they are.

**Response:** the three articles were identified in Google Scholar during the scoping review and feasibility stage before the formal database search commenced. This has now been clarified on page 11.

2. In the first description table of patients, it would be useful to have some basic demographic data. For example: age, sex.

**Response:** participants’ age and sex have been added to Table 2 (pages 13-15).

**Reviewer #2:**

The authors conducted a systematic review and meta-analysis of 10 studies to determine the association between stress during hospitalization and outcomes. Although the review is based on a limited number of studies, the results are interesting because the psychological stress patients feel during their hospital stay is associated with adverse outcomes, especially in-hospital and subjective outcomes. In addition, the reviewer believes that the article presents a significant finding because such studies still need to be done more. The reviewer also finds the paper well-written, including the strengths and limitations of the study. A minor comment is that the studies included in the systematic review need to indicate which disease treatments.

**Response:** participants’ condition/treatment have been added to Table 2 (pages 13-15).
